# Fuzzy Fault-Tolerant Following Control of Bionic Robotic Fish Based on Model Correction

**DOI:** 10.3390/biomimetics10080548

**Published:** 2025-08-20

**Authors:** Yu Wang, Jian Wang, Huijie Dong, Di Chen, Shihan Kong, Junzhi Yu

**Affiliations:** 1 The School of Intelligence Science and Technology, The Institute of Artificial Intelligence, University of Science and Technology Beijing, Beijing 100083, China; yuwang@ustb.edu.cn (Y.W.); di.chen@ustb.edu.cn (D.C.); 2 The Laboratory of Cognitive and Decision Intelligence for Complex System, Institute of Automation, Chinese Academy of Sciences, Beijing 100190, China; jianwang@ia.ac.cn; 3 The Key Laboratory of Advanced Transducers and Intelligent Control System, Ministry of Education, Taiyuan University of Technology, Taiyuan 030024, China; donghuijie@tyut.edu.cn; 4 The State Key Laboratory for Turbulence and Complex Systems, Department of Advanced Manufacturing and Robotics, College of Engineering, Peking University, Beijing 100871, China; kongshihan@pku.edu.cn

**Keywords:** bionic underwater robot, fault-tolerant control, fuzzy control, model correction

## Abstract

Fault-tolerant control for bionic robotic fish presents significant challenges due to the complex dynamics and asymmetric propulsion introduced by joint failures. To address this issue, this paper proposes a fault-tolerant following control framework for multi-joint bionic robotic fish by combining fuzzy control methodologies and dynamic model correction. Firstly, offline fault analysis is conducted based on the dynamic model under multi-variable parameter conditions, quantitatively deriving influence factor functions that characterize the effects of different joint faults on velocity and yaw performance of the robotic fish. Secondly, an adaptive-period yaw filtering algorithm combined with an improved line-of-sight navigation method is employed to accommodate the motion characteristics of bionic robotic fish. Thirdly, a dual-loop following control strategy based on fuzzy algorithms is designed, comprising coordinated velocity and yaw control loops, where velocity and yaw influence factors serve as fuzzy controller inputs with expert experience-based rule construction. Finally, extensive numerical simulations are conducted to verify the effectiveness of the proposed method. The obtained results indicate that the bionic robotic fish can achieve fault-tolerant following control under multiple fault types, offering a valuable solution for underwater operations in complex marine environments.

## 1. Introduction

Ocean resources are abundant, including mineral resources, biological resources, and renewable energy sources, characterized by significant development potential and high strategic value. With the continuous advancement of human exploration in deep-sea environments, increasingly stringent demands are placed on efficient, safe, and intelligent underwater operational equipment. Consequently, underwater robotics has become an indispensable technology for the development of marine resources. Traditional underwater vehicles predominantly employ propeller propulsion systems, which offer significant advantages in terms of thrust generation and high-speed locomotion capabilities. However, these conventional systems also present certain limitations including higher noise levels and reduced maneuverability in confined spaces [1,2]. In recent years, benefitting from advances in bionic engineering and sophisticated control theory, bionic robotic fish that mimic natural fish swimming patterns have gradually emerged as a prominent research direction in underwater robotics [3,4,5]. Bionic robotic fish achieve propulsion through tail fin oscillation, offering distinct advantages including superior maneuverability. Biomimetic robotic fish with enhanced maneuverability are particularly valuable for challenging underwater applications, including autonomous exploration in complex obstacle-rich environments, inspection missions in narrow underwater infrastructure passages, and biological monitoring in delicate ecosystems where minimal disturbance is required.

With respect to underwater resource acquisition missions, accurate path-following control constitutes a fundamental component in ensuring operational reliability and task completion efficiency. To achieve high-precision navigation and motion regulation, substantial research efforts have been dedicated to address path-following control challenges in underwater robotic systems [6,7,8]. However, during mission execution in complex underwater environments, underwater robots may encounter various structural or control-related malfunctions, which can significantly compromise their motion performance and mission execution capabilities. To this end, fault-tolerant control (FTC) strategies for conventional propeller-driven underwater vehicles have been relatively well-established [9,10,11,12,13].

**Figure 1:**
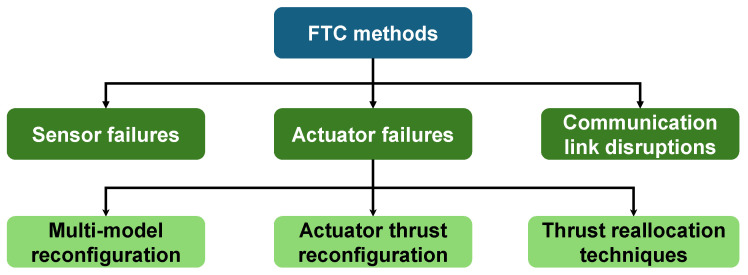
FTC methods illustration.

Depending on the failure modes, they can typically be categorized into three distinct types: actuator failures, sensor failures, and communication link disruptions [14], as shown in Figure 1. Among these categories, actuator fault-tolerant control constitutes a more thoroughly investigated domain, predominantly addressing thruster force degradation or rudder surface jamming [14,15,16]. Existing methods are generally classified into three primary categories. The first category involves multi-model reconfiguration, which establishes multiple predefined fault models with corresponding controllers and implements online switching to the most appropriate control law upon fault detection [17,18]. The second category focuses on actuator thrust reconfiguration strategies, which integrate dynamic models with advanced control algorithms by incorporating actuator fault severity levels into the control framework through thrust allocation modules [19,20,21]. The third category includes thrust reallocation techniques, which exploit system redundancy and employ methods to appropriately distribute generalized control forces [22]. Che developed a fault-tolerant control scheme for underactuated autonomous underwater vehicles (AUVs) with actuator faults by combining the backstepping method with single-criter network-based adaptive dynamic programming [23]. Li et al. proposed a robust adaptive fuzzy fault-tolerant control strategy for underactuated unmanned surface vessel (USV) formation tracking with multiple constraints and intermittent actuator faults, combining dynamic surface control with prescribed performance control techniques [24]. Chaos et al. presented a simple and robust fault-tolerant control scheme for AUVs under critical actuator failures, using only a single thruster with two control actions to guide the vehicle along a spiral-like path to a safe target point [25]. Jiang et al. investigated the fault-tolerant characteristics of a tripod parallel manipulator for underwater vectored thrusters through constraint screw theory analysis, and established an adaptive fault-tolerant control framework to handle actuator failures [26]. Wang et al. addressed time-varying thruster faults in autonomous underwater vehicles through an adaptive iterative learning observer that simultaneously estimated fault efficiency factors and overall disturbances for enhanced trajectory tracking control [27].

Compared to conventional AUVs, propulsion faults can exert considerably more negative effects on bionic robotic fish. Since robotic fish depend on coordinated oscillatory motion of multiple tail joints to achieve propulsion, the occurrence of locking or abnormal response in one or several joints can directly compromise the hydrodynamic configuration, resulting in diminished propulsive efficiency or complete locomotion failure. In particular, when critical propulsive joints experience malfunctions, the conventional tail-based propulsion mechanism becomes entirely ineffective, whereby the propulsive forces generated by subsequent joints are not only substantially attenuated but may also produce undesirable lateral forces and moments, imposing catastrophic impacts on velocity regulation and directional control. Consequently, the development of fault-tolerant control strategies specifically tailored for bionic propulsion failures becomes critically important. Research on fault-tolerant control methodologies for bionic robotic fish remains relatively limited in the current literature. Yang et al. developed a fault-tolerant control method for a bionic robotic fish with stuck tail joints, combining a central pattern generator (CPG)-based feedback controller with a feedforward compensator to maintain desired motion despite actuator damage [28]. Fan et al. proposed a sensor fault diagnosis method for a robotic fish, achieving successful fault diagnosis by converting sensor signals into fused images for classification [29]. Remmas et al. developed an active fault-tolerant control scheme for a bionic underwater robot with four flexible fins, using force allocation matrix reconfiguration to maintain motion control with three operational fins when actuator faults occur [30].

However, the above studies predominantly focused on simple fault condition or specific operational environments, lacking systematic investigations into following control for multi-joint bionic robotic fish under multiple fault types. The principal challenges encompass two critical aspects. First, faults in bionic robotic fish are primarily concentrated within the tail propulsion mechanism, which comprises multiple serially connected joints, where different joint failures exhibit significantly distinct dynamic response characteristics, necessitating control methodologies with substantial adaptive capabilities. Second, under fault types, the asymmetric nature of propulsive capacity may induce attitude disturbances and path deviations, imposing elevated requirements for coordination and robustness in control strategies. To this end, this paper focuses on the underwater following control tasks under fault types, aiming to provide a fault-tolerant following control framework for a robotic fish. The main contributions of this paper can be summarized as follows:

- Aiming to the requirements for underwater following control under fault types, a novel fault-tolerant following control framework is proposed for multi-joint bionic robotic fish. This framework successfully achieves precise following control under various fault scenarios by integrating dynamic model correction with fuzzy control methodologies.
- Based on the established dynamic model and the CPG model, systematic offline fault analysis is conducted under multi-variable parameter conditions, quantitatively obtaining influence factor functions of different joint faults and motion parameters on the velocity and yaw performance of the robotic fish, providing precise correction parameters for subsequent following controller design.
- A dual-loop following control strategy based on fuzzy algorithms is designed, comprising velocity and yaw controllers. The velocity influence factor and yaw influence factor are employed as inputs to the fuzzy controller, and a comprehensive fuzzy rule base is constructed based on expert experience. Additionally, considering the motion characteristics of bionic robotic fish, an adaptive period yaw filtering algorithm is developed, which, combined with an improved LOS navigation method, effectively achieves fault-tolerant following control under fault types. The results of extensive numerical simulation validate the effectiveness of the proposed algorithm.

The structure of this paper is organized as follows. Section 2 presents an overview of the problem formulation and the proposed control architecture. Section 3 conducts comprehensive fault characterization utilizing the developed dynamic model. Section 4 provides a detailed description of the fault-tolerant following control methodologies. Subsequently, Section 5 demonstrates the simulation results accompanied by thorough performance analysis. Finally, Section 6 summarizes the research and outlines future work.

## 2. Problem Statement and Control Framework

**Figure 2:**
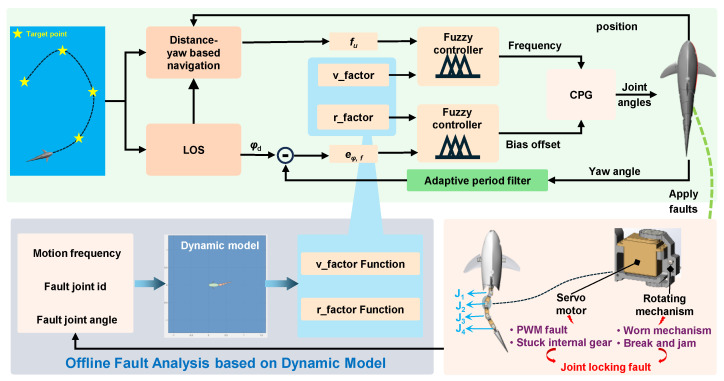
The schematic diagram of fault-tolerant following control for bionic robotic fish.

When a bionic robotic fish carries out cruising and exploration tasks in designated water areas, it typically requires precise path following along a reference path composed of predefined navigation points. However, if the caudal propulsion mechanism results in faults, the fish’s propulsion capability is significantly reduced, which can severely compromise the effectiveness of task execution. To address this issue, we propose a fault-tolerant control framework for robotic fish that integrates model correction with fuzzy control, as illustrated in Figure 2. The framework adopts a hierarchical control strategy, decomposing the path following task into two interrelated subsystems: a speed control loop and a yaw control loop.

- Speed control loop: This loop is primarily responsible for adjusting the swimming speed of the robotic fish to achieve the desired forward propulsion. Initially, a distance-yaw-based navigation algorithm is employed to calculate the spatial distance between the current location and the target point. Combined with yaw angle, multiple forms of the approach control factor $f_{u}$ are designed and input into the fuzzy controller. Based on $f_{u}$ and a designed fuzzy rule, the fuzzy controller dynamically adjusts the oscillation frequency of the CPG network, thereby modulating the flapping frequency of the tail. When the robotic fish is far from the target, the system increases the flapping frequency to enhance propulsion speed. As it approaches the target, the frequency is appropriately reduced to enable smooth deceleration and precise arrival.
- Yaw control loop: This control loop is specifically designed to manage the heading regulation of the robotic fish. The system employs the LOS guidance method to compute the desired heading angle $\psi_{d}$, which is then compared to the current yaw angle to obtain the filtered heading error $e_{\psi ,f}$. Subsequently, $e_{\psi ,f}$ is input into the fuzzy controller, which outputs a bias offset signal to adjust the asymmetric flapping amplitude of the caudal fin. This generates a steering torque that corrects the heading and enables the robotic fish to follow the target accurately. In addition, the system incorporates an adaptive period filter that dynamically adjusts its filter parameters based on the current swimming frequency. This module can effectively enhance the system’s resistance to disturbances and improve control stability.

More importantly, the proposed control framework introduces dedicated correction factors for each control loop, namely the speed influence factor $v_{factor}$ and the yaw influence factor $r_{factor}$. These two key parameters are obtained through an offline analysis based on a high-fidelity dynamic model. The procedure for acquiring these influence factors is as follows: First, a typical failure scenario, i.e., joint lock-up, is artificially imposed in an established dynamic model. Then, a series of systematic offline simulations are conducted across a broad parameter space to collect fault response data. This dataset captures the system’s translational and rotational velocity responses under varying conditions, including different faulty joint indices $joint_{i}\;\left(i=1,2,3,4\right)$, i.e., $J_{i}$, tail frequencies freq, and locked joint angles $angle_{f}$. Based on statistical analysis and function fitting of extensive simulation data, the following mapping relationships for influence factors can be established as follows:

(1) $$\begin{array}{c} v_{factor}=g_{u}\left(joint_{i},\;freq,\;angle_{f}\right)\\ r_{factor}=g_{r}\left(joint_{i},\;freq,\;angle_{f}\right) \end{array},$$

where $g_{u}\left(\cdot \right)$ and $g_{r}\left(\cdot \right)$ represent discrete data-driven function libraries for the speed and yaw influence factors, respectively. These mappings quantitatively characterize the extent to which specific fault types affect the locomotion performance of the robotic fish. It should be noted that both $v_{factor}$ and $r_{factor}$ represent steady-state statistical parameters derived from systematic parametric studies, where only the steady-state response data from multiple dynamic simulations are collected and analyzed, making these factors time-independent empirical correlations. During real-time control, the system promptly queries the corresponding influence factor values based on the current failure state. These values are subsequently used as input parameters for fuzzy controllers. By dynamically adjusting the input of fuzzy rules and calculating the output, the control system can precisely compensate for altered dynamics caused by failures. This model correction approach can effectively bridge the gap between theoretical control algorithms and real-world fault responses, offering a crucial foundation for ensuring the reliable operation of bionic robotic fish in complex and failure-prone environments.

## 3. Fault Analysis Based on Dynamic Model

### 3.1. Fault Type Illustration

**Figure 3:**
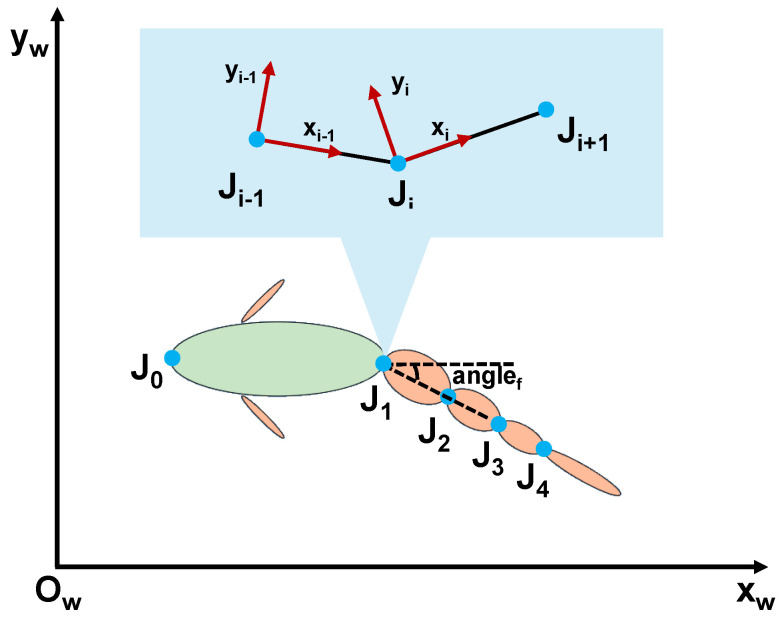
Fault illustration and coordinate system.

Joint locking fault represents a common type of mechanical failure encountered during the actual operation of bionic robotic fish, characterized by the loss of normal motion capability in specific joints, which become fixed at a particular angular position and are unable to continue executing oscillatory movements. As illustrated in Figure 3, when a joint lock fault occurs in any of the robotic fish joints (taking $J_{1}$ as an example), the affected joint remains locked at the moment of fault occurrence, completely losing its responsiveness to control commands and becoming incapable of performing subsequent periodic oscillatory motions. This fault mode directly impacts the propulsive performance and maneuverability of the robotic fish, posing significant challenges to its motion control system.

Regarding the fault mechanisms, analysis from the perspective of the drive system reveals that joint locking faults typically originate from multiple potential failure points within the servo motor system. First, abnormalities or interruptions in pulse-width modulation (PWM) control signals may cause the motor to lose normal driving force, yet since the motor power supply remains intact, the motor exhibits a locked condition. Second, wear, jamming, or poor lubrication in the worm gear mechanism can also result in mechanical blockage of the transmission chain. Additionally, issues such as loosening, fracture, or foreign object obstruction in the gear assembly and belt transmission system may also trigger complete joint locking. These fault modes often exhibit sudden onset characteristics, which are difficult to prevent through conventional maintenance measures. It is noteworthy that, beyond locking faults, another disabled failure mode exists where robotic fish joints exhibit free oscillation. This type of fault primarily results from loss of power supply to the control system (such as poor contact or wire breakage), etc. For multi-joint bionic robotic fish employing series-connected joint configurations, the power and ground lines of all four motors are typically consolidated into a single channel to save wiring space, then uniformly connected to the battery system. Consequently, power failure tends to cause simultaneous loss of control capability across all joints. Based on this consideration, this paper focuses primarily on joint locking faults and does not address scenarios involving disabled multi-joint failure.

### 3.2. Motion Model of the Bionic Robotic Fish

To accurately simulate the motion characteristics of a bionic robotic fish under fault types, we construct a motion model grounded in multi-joint dynamics theory. The robotic fish is abstracted as a serial structure consisting of a head joint followed by a four-link tail mechanism, as shown in Figure 3. Dynamic analysis is performed using the Newton–Euler formulation, and the details can be found in our previous work [31]. First, an inertial coordinate system is established as $C_{w}=\left(o_{w},x_{w},y_{w},z_{w}\right)$. For each segment of the robotic fish body, a corresponding body-fixed coordinate system is defined as $C_{i}=\left(o_{i},x_{i},y_{i},z_{i}\right)$. Here, $L_{0}$ refers to the head segment, and its origin $o_{0}$ is located at the foremost point of the fish. For the remaining segments, the origin $o_{i}$ of each local coordinate system coincides with the center of $J_{i}$. The rotational displacement at $J_{i}$ is denoted by $\theta_{i}$, where $\theta_{0}$ represents the yaw angle.

Based on the kinematic analysis of serial multi-joint systems, the velocity propagation between adjacent body segments can be described using the following recursive relation:

(2) Vi=UiΩi=Hi−1  iVi−1+θ˙iKi,

where $V_{i}={\left[U_{i}^{T},\Omega_{i}^{T}\right]}^{T}$ represents the velocity vector of each link expressed in the local coordinate frame $C_{i}$, where $U_{i}$ and $\Omega_{i}$ are the linear and angular velocity vectors, respectively. $K_{i}$ denotes the joint axis vector, and Hi−1  i is the homogeneous transformation matrix. Finally, by applying the Newton–Euler method, the dynamic equation for each link is formulated as follows:

(3) Gi−1,i  i=MiVi+γi−Fi+Hi+1i+1iGi,i+1,

where $M_{i}$ represents the inertia matrix, and $\gamma_{i}$ and $F_{i}$ denote the Coriolis and hydrodynamic terms, respectively. Gi−1,i  i indicates the constraint force between adjacent links.

This dynamic model establishes a nonlinear mapping between the joint motion states $\left(\theta_{i},{\dot{\theta }}_{i},{\ddot{\theta }}_{i}\right)|i\in \left[1,n\right]$ and the global motion state $V_{0}$ of the robotic fish.

Furthermore, the joint motion states are generated using a CPG framework, which is governed by neural structures known as CPG. The CPG model adopted in this work is formulated as follows [32]:

(4) $$\left\{\begin{array}{c} {\dot{x}}_{i}=-\omega_{i}\left(y_{i}-b_{i}\right)+x_{i}\left(r_{i}^{2}-x_{i}^{2}-{\left(y_{i}-b_{i}\right)}^{2}\right)+h_{1}\left(x_{i-1}\cos\varphi_{i}+\left(y_{i-1}-b_{i-1}\right)\sin\varphi_{i}\right)\\ {\dot{y}}_{i}=\omega_{i}x_{i}+\left(y_{i}-b_{i}\right)\left(r_{i}^{2}-x_{i}^{2}-{\left(y_{i}-b_{i}\right)}^{2}\right)+h_{2}\left(x_{i+1}\sin\varphi_{i+1}+\left(y_{i+1}-b_{i+1}\right)\cos\varphi_{i+1}\right) \end{array}\right.,$$

where (i=1,…,n), n=4 denotes the number of CPG units. The variables $x_{i}$ and $y_{i}$ represent the state variables of the system. The parameters $\omega_{i}$ and $r_{i}$ denote the intrinsic frequency and amplitude of the *i*-th oscillatory neuron, respectively, where $r_{i}=8{}^{\circ},16{}^{\circ},24{}^{\circ},32{}^{\circ}$ for different oscillatory neurons and $\omega_{i}$ is set according to different swimming requirements. The term $\varphi_{i}=30{}^{\circ}$ represents the phase difference between adjacent oscillators. $h_{1}$ and $h_{2}$ are coupling gains, which are set as 1. $b_{i}$ denotes the bias offset for turning motion and serves as the control variable.

### 3.3. Offline Analysis Under Different Fault Types

Based on the aforementioned fault analysis and dynamic model, this section conducts offline fault data collection based on the dynamic model to construct the lookup table for Equation (1). By setting tail oscillation frequencies $freq\in \left[0.4,1.5\right]$ Hz and locked joint angles $angle_{f}\in \left[-40{}^{\circ},40{}^{\circ}\right]$, systematic simulations were performed for different joints.

**Figure 4:**
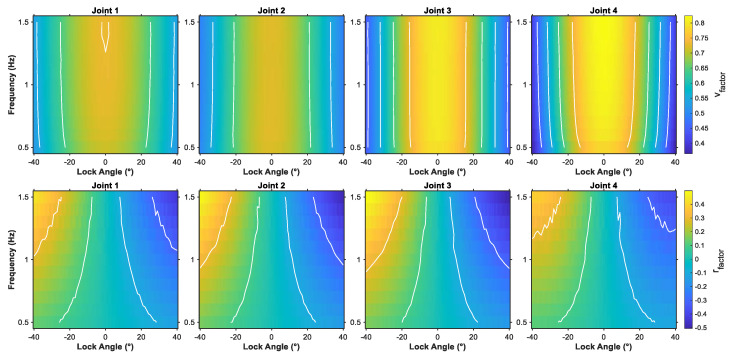
Impact of joint locking faults on velocity and yaw performance for the robotic fish.

Figure 4 illustrates the variation patterns of velocity influence factor $v_{factor}$ and yaw influence factor $r_{factor}$ when locking faults affect different joints under various locked angles and frequencies. Moreover, we add appropriate contour lines to highlight key threshold regions in areas of extreme values. Analysis of the velocity influence factor heat map reveals that, regardless of which joint is affected by the locking fault, $v_{factor}$ exhibits a distinct symmetric distribution characteristic. Velocity loss is minimal when the locked angle approaches 0°, and the velocity impact gradually intensifies as the absolute value of the locked angle increases. Furthermore, while the influence trends across different joints remain generally consistent, $J_{4}$ demonstrates slightly higher negative impact on velocity under large-angle locking conditions compared to other joints, suggesting that tail motion freedom contributes more significantly to propulsive performance. The modulation effect of CPG frequency on velocity influence is relatively weak, with trends remaining essentially consistent across different frequencies, showing only slight improvement under high-frequency conditions. In the heat map of yaw influence factor $r_{factor}$, an overall center-symmetric distribution characteristic emerges with respect to locked angle variations. The $r_{factor}$ exhibits a negative correlation with the locked angle, with the locked angle directly determining both the yaw direction and extent of the robotic fish. The $r_{factor}$ distribution also exhibits significant differences among joints. $J_{1}$ and $J_{2}$ demonstrate higher sensitivity to yaw influence when locked, with $r_{factor}$ amplitude increasing significantly under large locked angle conditions, while $J_{3}$ and $J_{4}$ show relatively smaller overall yaw influence.

**Figure 5:**
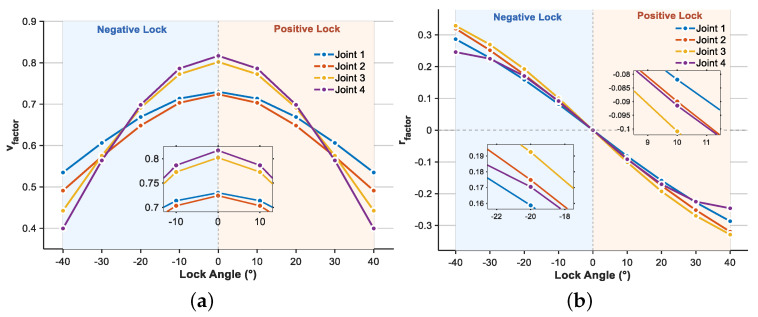
The fault factors illustration under different joints. (**a**) $v_{factor}$. (**b**) $r_{factor}$.

For further analysis, we fixed the swimming frequency at 1 Hz and focused on investigating the effects of different locked angles on the velocity influence factors of each joint, as shown in Figure 5a. The results demonstrate that $J_{1}$, $J_{2}$, $J_{3}$, and $J_{4}$ achieve $v_{factor}$ values of 0.730, 0.724, 0.802, and 0.817, respectively, at the 0° locked angle, representing the maximum values for each joint across the entire angular range. In contrast, under the −40° locked angle condition, the $v_{factor}$ values of the four joints decrease to their minimum levels: 0.535 ($J_{1}$), 0.491 ($J_{2}$), 0.443 ($J_{3}$), and 0.400 ($J_{4}$). Comparative analysis reveals that during the angular transition from −40° to 0°, the $v_{factor}$ improvement rates for the four joints are 36.4%, 47.5%, 81.0%, and 104.3%, respectively. This finding further confirms the critical role of the tail joint in the propulsive performance of robotic fish. Similarly, under the 1 Hz swimming frequency, the effects of different locked angles on the yaw influence factor $r_{factor}$ are shown in Figure 5b. Regarding the variation amplitude, $J_{3}$ demonstrates the largest $r_{factor}$ range of 0.658 (from 0.329 to −0.329), followed by $J_{2}$ with 0.640, while $J_{1}$ and $J_{4}$ show variations of 0.574 and 0.492, respectively.

## 4. Fuzzy-Based Following Control of Bionic Robotic Fish

Based on the results of the aforementioned fault-tolerant analysis, we conduct the following controller in this section. Considering the highly nonlinear dynamic characteristics of robotic fish, a fuzzy logic-based following control method is proposed, incorporating the velocity influence factor and yaw influence factor obtained from the fault-tolerant analysis as key input parameters for the fuzzy rule. Furthermore, through the improved design of line-of-sight navigation algorithm and adaptive yaw filtering module, robust following control of the bionic robotic fish under fault types is achieved.

### 4.1. Navigation Strategy Based on LOS

The LOS navigation method is widely applied in marine vehicle navigation due to its simplicity and effectiveness. Hence, we employ an LOS navigation strategy incorporating a look-ahead distance mechanism. This approach projects a virtual reference point along the vehicle’s current heading direction rather than directly calculating the navigation angle from the current position to the target point. Specifically, given the current state vector of the robotic fish as (x,y,ψ), where (x,y) represents the position coordinates and ψ denotes the current heading angle, and the target waypoint as $\left(x_{t},y_{t}\right)$, the calculation formula for the virtual look-ahead point $\left(x_{v},y_{v}\right)$ is $x_{v}=x+L_{vh}\cos\left(\psi \right)$, $y_{v}=y+L_{vh}\sin\left(\psi \right)$, where $L_{vh}$ denotes the predefined look-ahead distance. Subsequently, the virtual LOS angle from the forward-looking point to the target point can be calculated as follows:

(5) $$\psi_{los}=\arctan\left(\frac{y_{v}-y_{t}}{x_{v}-x_{t}}\right).$$

Furthermore, the yaw error input to the yaw controller can be defined as $e_{\psi }=\psi_{los}-\psi$. The introduction of look-ahead distance $L_{vh}$ provides several advantages. On one hand, it acts as a low-pass filter, attenuating high-frequency oscillations in guidance commands and enhancing system stability. On the other hand, it enables the vehicle to initiate heading corrections earlier, generating smoother trajectories and reducing overshoot.

### 4.2. Adaptive Period Filter for Yaw Angle

For bionic robotic fish, due to the unique propulsion mechanism, the yaw angle signal typically contains periodic oscillatory components caused by tail fin undulation, resulting in oscillatory characteristics in control outputs and severely degrading the accuracy and stability of yaw control. Therefore, it is crucial for achieving high-quality yaw control to design effective filtering methods to extract the trend component of yaw angle and suppress oscillatory noise.

Let the original yaw angle error signal be $e_{\psi ,raw}\left(t\right)$, and this signal can be decomposed into

(6) $$e_{\psi ,raw}\left(t\right)=e_{\psi ,dc}\left(t\right)+e_{\psi ,osc}\left(t\right),$$

where $e_{\psi ,dc}\left(t\right)$ represents the low-frequency trend component reflecting the actual heading deviation, and $e_{\psi ,osc}\left(t\right)$ denotes the periodic oscillatory component induced by tail fin undulation. The characteristics of the oscillatory component are closely related to the tail fin undulation frequency freq, with an oscillation period of T=1/freq that varies dynamically with the robotic fish’s motion state. To effectively extract the trend component $e_{\psi ,dc}\left(t\right)$, this paper applies a moving average filtering method based on adaptive window length, whose core principle is to dynamically adjust the filtering window length according to the current CPG undulation frequency to match the oscillation period, thereby maximizing the suppression of periodic oscillations. The specific process is shown in Algorithm 1.
**Algorithm 1** Adaptive period-based yaw angle filtering algorithm**Input:** Current CPG oscillation frequency freq, raw yaw error $e_{\psi ,raw}$, control period Δt **Output:** Filtered yaw error $e_{\psi ,f}$   1: Initialize persistent variables: yaw_error_buffer, buffer_idx, buffer_valid, last_N   2: Calculate oscillation period: T = max(1/freq,Δt)   3: Calculate window length: N = max(1,round(T/Δt))   4: **if** first run or buffer uninitialized **then**   5:    yaw_error_buffer$\;=\;e_{\psi ,raw}\;\times \;1_{N\;\times \;1}$   6:    buffer_idx = 0, buffer_valid = 0, last_N = N   7: **end if**   8: **if** last_N≠N **then**   ▹Window length changed, buffer reconstruction needed   9:    Save old buffer: old_buffer = yaw_error_buffer 10:    Save old parameters: old_idx = buffer_idx, old_valid  = buffer_valid 11:    Create new buffer: yaw_error_buffer$\;=\;0_{N\;\times \;1}$ 12:    **if** N< old_valid **then**                ▹ Window shrinking 13:        **for** k = 1 to *N* **do** 14:           idx = mod(old_idx−N+k−1, last_N) + 1 15:           yaw_error_buffer[k] = old_buffer[idx] 16:        **end for** 17:        buffer_valid = N, buffer_idx = N 18:    **else**                         ▹Window expanding 19:        **for** k = 1 to old_valid **do** 20:           idx = mod(old_idx − old_valid + k−1, last_N) + 1 21:           yaw_error_buffer[k] =  old_buffer[idx] 22:        **end for** 23:        **if** old_valid>0 **then** 24:           fill_value = old_buffer[mod(old_idx−1, last_N) + 1] 25:        **else** 26:           fill_value$\;=\;e_{\psi ,raw}$ 27:        **end if** 28:        yaw_error_buffer[old_valid + 1:N] = fill_value
 29:        buffer_valid = old_valid, buffer_idx = old_valid 30:    **end if** 31:    last_N = N 32:**end if** 33:Update circular buffer index: buffer_idx = mod(buffer_idx,N) + 1 34:Store new data: yaw_error_buffer[buffer_idx$]\;=\;e_{\psi ,raw}$ 35:Update valid data length: buffer_valid = min(buffer_valid + 1,N) 36:Calculate moving average: $e_{\psi ,mean}=\frac{1}{\mathrm{buffer\_valid}}\sum_{k=1}^{\mathrm{buffer\_valid}}\;\mathrm{yaw\_error\_buffer}\left[k\right]$ 37:Saturation limiting: $e_{\psi ,f}\;=\;\mathrm{sat}\left(e_{\psi ,mean},e_{\psi ,max}\right)$ 38:**return** $e_{\psi ,f}$

### 4.3. Fuzzy Controller Design

Based on the dynamic model, we obtain the effects of different faults on the velocity and yaw influence factor of the bionic robotic fish and constructed a corresponding mapping library. How to apply this mapping library as prior knowledge to fault-tolerant control is a key issue. Considering the highly nonlinear characteristics of the bionic robotic fish, this paper proposes a fault-tolerant control strategy based on the fuzzy method, using velocity and yaw velocity influence factors as inputs to the fuzzy rule, integrating prior model correction knowledge into the control process. Fuzzy inference comprises three steps: fuzzification, fuzzy rule design, and defuzzification. Fuzzification aims to determine the basic universes of discourse for input and output variables. We divide following control into velocity control loop and yaw control loop, designing fuzzy controllers for each, both adopting a dual-input single-output structure. For the velocity controller, the inputs are velocity influence factor $v_{factor}$ and approaching control factor $f_{u}$ (both dimensionless parameters). The output is the tail mechanism’s oscillation frequency (Hz). Based on the offline analysis from fault-tolerant modeling, the domain for velocity influence factor is set to [0, 1]. For the approaching control factor, we design three forms:

(7) $$f_{u}=\left\{\begin{array}{c} {\bar{e}}_{d}\\ \min\left({\bar{e}}_{d},1-{\bar{e}}_{yawf}\right)\\ {\left(1+e^{\left(-\frac{{\bar{e}}_{d}}{{\bar{e}}_{yawf}+\varepsilon }\right)}\right)}^{-1} \end{array}\right.,$$

where e¯d=ede¯d=edesgesg and e¯yawf=eψ,fe¯yawf=eψ,feψ,fmaxeψ,fmax. $e_{d}$ denotes the distance between the robotic fish’s real-time position and the current target point. $e_{sg}$ represents the distance between the starting point and the target point, and $e_{\psi ,fmax}$ indicates the preset upper limit of yaw angle error (50°). The first form (Mode 1) solely considers the normalized distance value between the robotic fish’s current position and target position. The second control mode (Mode 2) implements a minimum value strategy that integrates position error and yaw angle compensation. In this approach, position factors are prioritized when the robotic fish is in proximity to the target location, whereas attitude adjustment becomes dominant when significant yaw angle errors are detected. The third form (Mode 3) adopts an adaptive fusion strategy, calculating the ratio of position error to yaw angle error and mapping it to the interval [0, 1]. When position error is relatively large compared to yaw angle error (i.e., the robotic fish is far from the target but properly oriented), the output value approaches 1, indicating that CPG oscillation frequency should be increased for rapid target approach. Conversely, when yaw angle error is relatively large (i.e., the robotic fish is close to the target but significantly misoriented), the output value approaches 0, suggesting frequency reduction for precise attitude adjustment. The parameter ε is employed to prevent division-by-zero errors. Considering the limitation of the oscillation frequency of the robot fish, the domain of freq is set to [0.4, 1.5] Hz.

For the yaw controller, the inputs include yaw influence factor and yaw error, with the output being the CPG bias of the propulsive mechanism. Based on the offline data formed through fault-tolerant modeling analysis, the domain for the yaw influence factor can be set to [−0.4, 0.4], while the yaw error factor is range-limited to [−0.873, 0.873] rad based on the actual error. For the output bias of the yaw controller, we set its domain to [−0.698, 0.698] rad.

Furthermore, the design of fuzzy subsets corresponding to each state variable is also crucial. To enable the designed fuzzy inference system to achieve superior approximation performance, for the velocity controller, we divide the velocity influence factor, approaching control factor, and oscillation frequency into four fuzzy subsets each: ZE, PS, PM, PB. For the yaw controller, the yaw influence factor, yaw error factor, and oscillation bias are divided into seven fuzzy subsets: NB, NM, NS, ZE, PS, PM, PB. The membership functions for all the aforementioned linguistic variables are selected as either triangular membership functions or trapezoidal membership functions. The design of the fuzzy rule base constitutes a key step in the fuzzy inference algorithm, primarily consisting of data and fuzzy linguistic rules. This section employs “IF-THEN” fuzzy language to establish the fuzzy rule bases as shown in Table 1 and Table 2. Finally, by weighting and averaging the membership functions of the output variables, the final defuzzification process is carried out to obtain the final control quantity.

## 5. Simulation and Analysis

In this section, extensive simulations are conducted to validate the effectiveness of the proposed fault-tolerant following control method for bionic robotic fish. A comprehensive simulation environment is constructed in Matlab based on the established dynamic model to replicate realistic underwater conditions. To thoroughly evaluate the control performance, three representative following scenarios are implemented: waypoint following, pentagram waypoint following, and figure-eight path following. These tests are performed under various fault types to assess the robustness and adaptability of the proposed approach. Additionally, the advantages of the improved LOS guidance and adaptive filtering techniques are specifically examined through the waypoint following experiments to demonstrate their individual contributions to the overall control performance.

### 5.1. Results and Analysis for Waypoint Following Control

**Figure 6:**
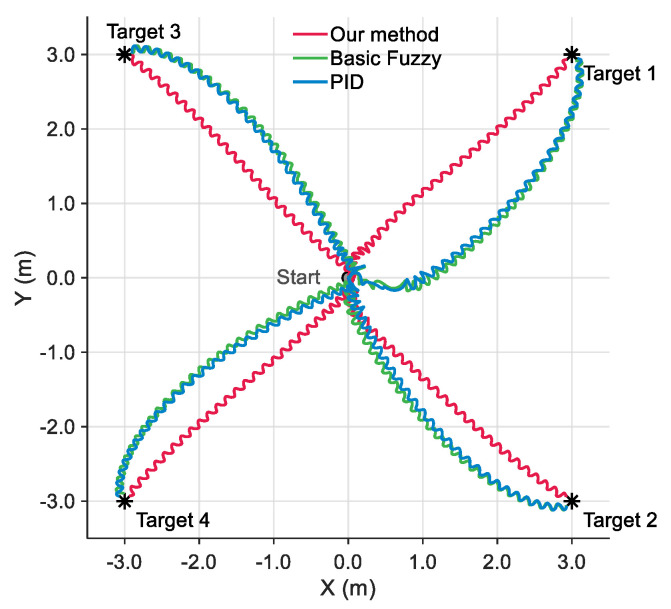
Path results of waypoint following control under different methods.

Figure 6 presents trajectory comparison results of the robotic fish navigating from the origin to target points in four quadrants under the fault type of $J_{4}$ locked at 40°. To validate the effectiveness of the proposed method, three comparative approaches are implemented. The Basic Fuzzy method does not apply velocity and yaw influence factors as inputs, relying solely on distance to destination and yaw angle error for feedback. The proportion–integration–differentiation (PID) method adopts a similar control strategy.

From trajectory analysis, the proposed method demonstrates superiority. Firstly, it achieves the smoothest and shortest motion paths. Secondly, it exhibits better convergence characteristics when reaching all target points, reflecting superior control precision and stability. In contrast, simulation results show that both Basic Fuzzy and PID methods exhibit obvious backward motion during the initial phase. This occurs since the robotic fish’s propulsion capability is limited under fault types. Conventional control methods lack prior knowledge and compensation mechanisms for fault effects, resulting in initial control commands that fail to generate effective forward thrust. Consequently, these two methods result in lengthened overall motion paths. In addition, the consistent performance across the four different quadrant tests demonstrates that the proposed method possesses good adaptability to target positions and motion directions.

**Figure 7:**
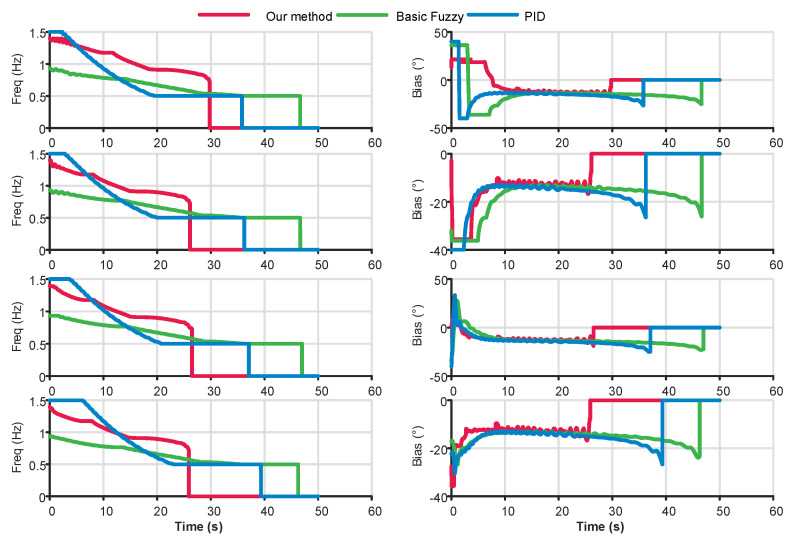
Control outputs results of waypoint following control under different methods.

Figure 7 presents the simulation results of the freq and Bias control signals. First, we examine the time at which the control signals decay to zero, which corresponds to the time of reaching the target points. The proposed method reaches the four target points at 29.78 s, 26.11 s, 26.49 s, and 25.88 s, respectively. In contrast, the Basic Fuzzy method requires 35.79 s, 36.23 s, 37.09 s, and 39.33 s, while the PID method takes 46.64 s, 46.66 s, 46.98 s, and 46.37 s. These results indicate that the proposed method achieves a performance improvement of 16% to 34% over the basic Fuzzy method, and 36% to 44% over the PID method. Regarding the bias output signals, all three methods are capable of generating correct control responses based on system feedback, with the proposed method demonstrating relatively superior responsiveness. However, it is worth noting that slight oscillations are observed in the bias signal of the proposed method. This phenomenon is mainly due to the fact that $r_{factor}$ is a multi-variable function. Although the joint ID and locking angle remain constant during operation, the swimming frequency varies in real-time, inducing $r_{factor}$ fluctuations and consequently introducing variations in the control output.

In addition, the yaw control accuracy for different target points was quantitatively evaluated. Table 3 lists the root-mean-square error (RMSE) of the filtered yaw error angles. The results indicate that the proposed method consistently achieves the smallest steady-state errors across all target points, further validating its effectiveness and superiority.

**Figure 8:**
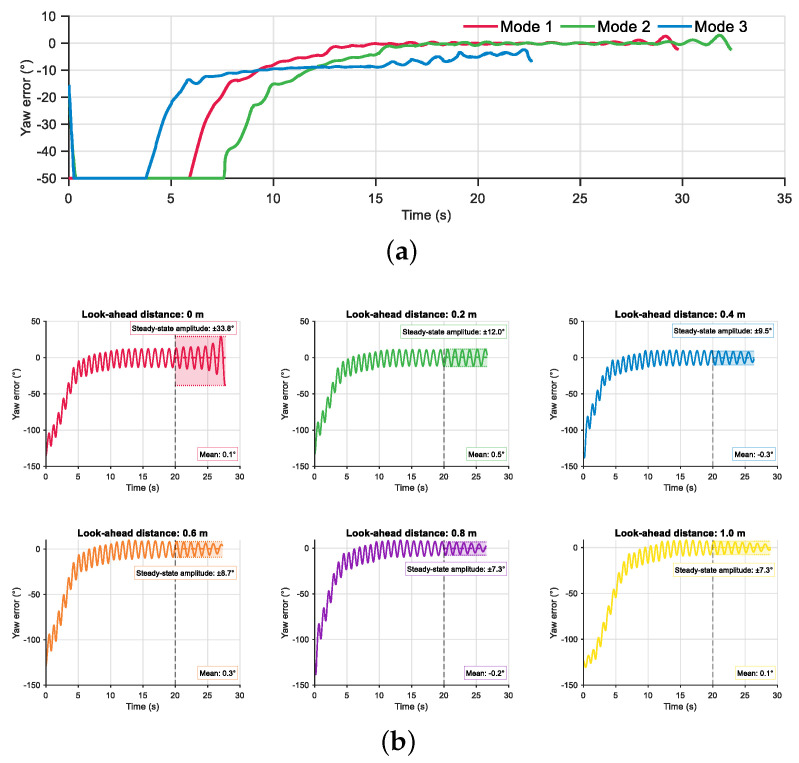
The comparison of different simulation setup. (**a**) Different $f_{u}$. (**b**) Different look-ahead distance.

Furthermore, to investigate the impact of different input parameter configurations in the fuzzy rule base of the velocity controller on control performance, a comparative experiment was designed based on the parameter $f_{u}$. As shown in Figure 8a, the effectiveness of three input strategies (Mode 1, Mode 2, and Mode 3) was evaluated by analyzing the time-domain response of the filtered yaw angle error. In the simulation, the robotic fish was tasked with moving from the initial point (0, 0) to the target point (3, 3), while performing point following control under a fault constraint where the fourth joint was locked at 40°. The results indicate that the Mode 3 strategy exhibits the fastest convergence of filtered yaw angle error with upper and lower bound. However, due to its relatively high output frequency, the system exhibits a noticeable steady-state error near the target point. The Mode 2 strategy results in a distinct phase-wise control behavior. In the initial stage of motion, since both distance and yaw angle errors are large, the system prioritizes yaw correction. Once the yaw error stabilizes, the frequency shifts to primarily reference the distance error. Comprehensive analysis suggests that the Mode 1 strategy demonstrates more balanced control performance. Although its convergence time is longer, the system reaches a steady state more quickly. It is worth noting that all three control strategies exhibit varying degrees of oscillation as the robotic fish approaches the target point.

Based on this oscillation observation, additional simulations were conducted to optimize terminal control performance by adjusting the look-ahead distance parameter $L_{vh}$. The values of $L_{vh}$ were set to [0, 0.2, 0.4, 0.6, 0.8, 1.0] m, and the system responses under each setting were systematically analyzed. To clearly illustrate the oscillation, Figure 8b presents the unfiltered yaw angle error data without upper and lower bound. The obtained results show that, when $L_{vh}=0$ m, the system exhibits significant terminal oscillations, with a steady-state amplitude of ±33.8°. Introducing $L_{vh}=0.2$ m markedly suppresses the oscillations, reducing the amplitude to ±12.0°. As $L_{vh}$ increases, the oscillation amplitude continues to decrease. However, it is observed that the suppression effect saturates when $L_{vh}$ exceeds 0.8 m. Moreover, an excessively large look-ahead distance implies overly anticipatory control actions, which may compromise system convergence. Therefore, the value of $L_{vh}$ should be selected appropriately based on the task specific requirements.

### 5.2. Path Following Control Results Under Different Fault Types

#### 5.2.1. Pentagram Waypoint Following

**Figure 9:**
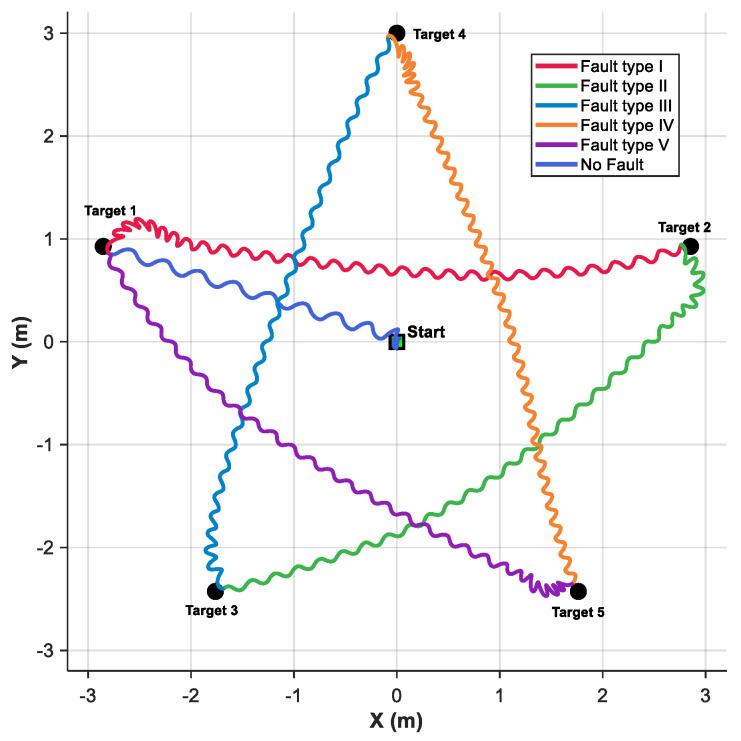
The path results of pentagram waypoint following under different fault types.

To evaluate the adaptability of the proposed method under various fault types, a pentagram waypoint following simulation was designed in this section. The simulation adopted Mode 1 input configuration for the velocity fuzzy rule and set the look-ahead distance to 0.2 m. The simulation results are illustrated in Figure 9. The robotic fish starts from the origin and sequentially passes through Target 1 to Target 5, ultimately forming a complete pentagram trajectory. The task consists of six following segments. The first segment (from the starting point to Target 1) is executed under fault-free conditions. Starting from Target 1, the system switches to a different fault mode at each subsequent target point. The specific fault configurations are detailed in Table 4. The primary challenge of pentagram waypoint following task lies in the requirement for large-angle turning maneuvers at each target point to accurately orient the robotic fish toward the next destination. The obtained results demonstrate that, under the proposed control method, the robotic fish successfully completes all segments. Furthermore, the impact of different fault types on the trajectory shape varies. For instance, faults in $J_{2}$ and $J_{3}$ cause more pronounced trajectory distortion, which aligns with the qualitative and above quantitative fault analyses. Despite the aforementioned influences, the deviations induced by each fault type are effectively mitigated through the proposed fault-tolerant control strategy, highlighting the method’s effectiveness in maintaining accurate following under adverse conditions.

**Figure 10:**
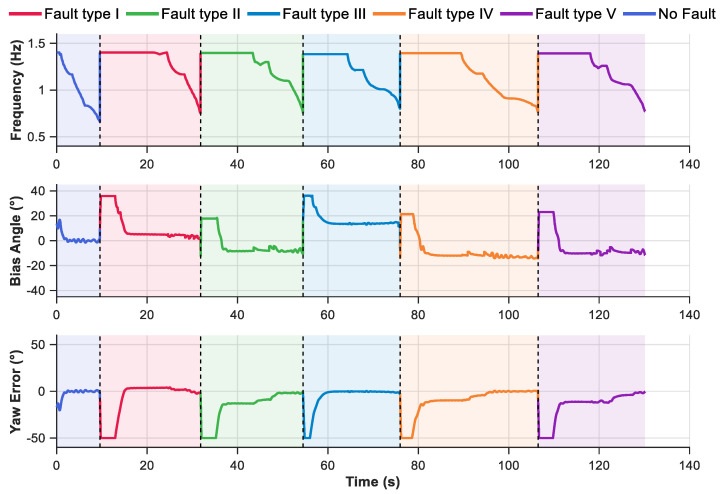
The results of control outputs and yaw error for pentagram waypoint following.

To further analyze the dynamic response characteristics of the system during pentagram waypoint following task, Figure 10 presents the time-domain response curves of the control frequency, bias angle, and filtered yaw error angle. The entire following process is divided into six segments, each marked with a different background color to distinguish between the fault-free condition and the five distinct fault modes. The bias angle result indicates that the system possesses a rapid adjustment capability during target point transitions, with all bias angles eventually converging to around 0°. It is important to note that a yaw error limit of ±50° was imposed to enhance the precision of fuzzy control. At the moment of fault mode switching, the system exhibits highly coordinated adjustment behavior. The control frequency and bias angle respond in near-synchrony, and transient fluctuations in yaw error are effectively suppressed within acceptable bounds. Therefore, despite the presence of different fault constraints, the control performance across all segments remains relatively consistent. This further validates the proposed method’s stability under multiple fault types. However, since the employed control strategy focuses on waypoint following rather than strict path following, minor deviations from the ideal straight-line trajectory are observed in some segments.

#### 5.2.2. Figure-Eight Path Following

**Figure 11:**
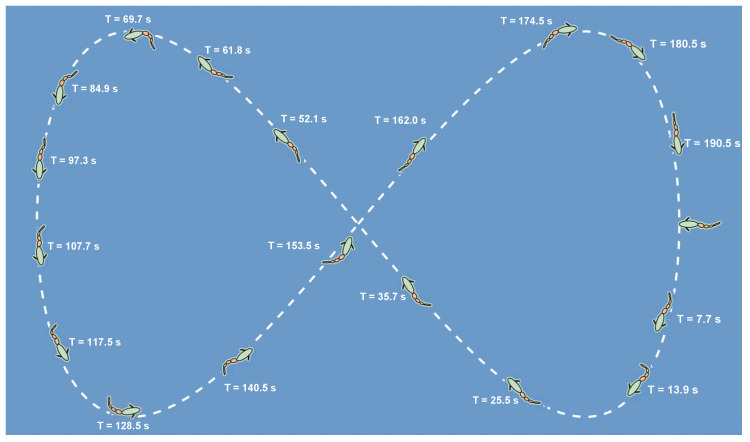
Snapshot sequences for figure-eight path following under different fault types.

**Figure 12:**
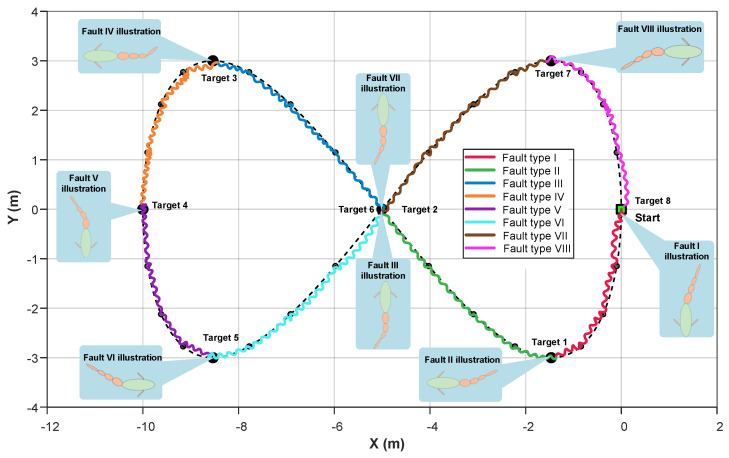
The path results of figure-eight path following with fault illustration.

To further investigate the path deviation observed during the aforementioned waypoint following process, an additional simulation was conducted using a figure-eight path. The resulting sequence diagrams and trajectory plots are shown in Figure 11 and Figure 12, respectively. In this simulation, the figure-eight path is defined by the following expression:

(8) $$\left\{\begin{array}{c} x_{ref}=5\sin\left(\alpha \right)-5\\ y_{ref}=3\sin\left(2\alpha \right) \end{array}\right.,$$

where $\alpha \in \left[0,2\pi \right]$. The robotic fish starts from the origin, with eight target points set, corresponding to α=nπnπ44(n=3,4,…,10), respectively.

Similarly, a fault mode transition was introduced at each target point during this simulation. A diverse set of fault configurations was applied, with graphical representations of the specific fault type shown in Figure 12. To achieve a smoother and more continuous path-following performance, three intermediate reference points were uniformly inserted between every two target waypoints. The simulation results indicate that, under the proposed control strategy, the robotic fish successfully completed the full figure-eight path-following task. The resulting trajectory closely matches the figure-eight curve. The introduction of intermediate reference points improved the smoothness and continuity of the following path. As illustrated in the fault diagrams, different fault types introduce considerable changes to the fish’s body configuration. Notably, although the system experienced eight different fault modes across the eight following segments, including various joint locking faults and combinations of different locking angles, the robotic fish consistently adapted quickly after each fault transition and maintained stable following performance. The system demonstrated particularly good performances near the intersection points of the figure-eight path and in regions with high curvature variation. The symmetry and geometric integrity of the overall trajectory were well preserved throughout the experiment, further confirming the practical applicability of the proposed fault-tolerant control algorithm.

### 5.3. Discussion

Bionic robotic fish demonstrate unique advantages in complex underwater environments due to their excellent maneuverability. However, their multi-joint propulsion mechanisms are prone to actuator faults when executing high-frequency and large-amplitude motions. Such faults significantly alter the system’s dynamic characteristics, severely impacting the motion performance of robotic fish. To address these issues, this paper proposes a fault-tolerant following control framework that integrates dynamic model correction with fuzzy control. By establishing a comprehensive dynamic model, we conduct systematic fault analysis, quantifying the influence patterns of different joint faults on the motion characteristics of robotic fish, thereby providing universal theoretical references for fault-tolerant control of multi-joint bionic robotic fish. The quantitative results demonstrate performance improvements of the proposed method. Convergence speed was enhanced by 16–34% over Basic Fuzzy and 36–44% over PID methods, while RMSE values were reduced by approximately 95%, representing substantial improvement in control accuracy. Additionally, terminal oscillations were effectively suppressed from ±33.8° to ±12.0° through look-ahead distance optimization, validating the practical effectiveness of the fault-tolerant control strategy. Compared with existing fault-tolerant following control methods, the distinctive feature of the proposed approach lies in organically incorporating the model prior knowledge of fault effects into the feedback control loop, rather than employing traditional fixed feedforward compensation strategies [28].

Although we successfully achieve fault-tolerant following control of bionic robotic fish under different fault types, there are some aspects that can be improved. First, the current research only considers joint lock-up failure modes. It should be noted that robotic fish systems are susceptible to various imperfections including circuit malfunctions, sensor degradations, and different actuator failure modes, some of which may introduce complex dynamical behaviors such as unexpected strange attractors. However, based on our laboratory experience with servo-controlled tail actuators, joint lock-up represents the most frequently encountered fault mode, which motivates our focus on this specific failure type. Beyond joint lock-up faults, other actuator failure modes such as disabled joint failures also warrant investigation. By introducing spring-damper dynamic models to describe the dynamic characteristics of disabled joint failures, this type of fault condition can be effectively simulated. Second, we do not consider the complex scenario of simultaneous failures in multiple joints. Considering the high-dimensional and nonlinear characteristics of such problems, reinforcement learning-based algorithms may be more effective.

## 6. Conclusions and Future Work

In this paper, we have developed a fault-tolerant following control framework for multi-joint bionic robotic fish. With regard to the fault analysis, influence factor functions were quantitatively derived based on the established dynamic model under multi-variable parameter conditions, characterizing the effects of different joint faults on velocity and yaw performance. Next, an adaptive-period yaw filtering algorithm combined with an improved LOS navigation method was employed to accommodate the motion characteristics of bionic robotic fish. Utilizing the derived influence factors as input states, a dual-loop following control strategy based on fuzzy algorithms was designed, comprising mutually coordinated velocity and yaw control loops with expert experience-based rule construction. The fuzzy control framework was integrated with dynamic model correction to enable precise following control under various fault scenarios.

In the future, we will focus on more fault categories of the robotic fish, thereby obtaining more comprehensive fault characterization. Furthermore, an adaptive learning-based control strategy that can automatically adjust to different fault patterns is another area worthy of investigation. Additionally, we plan to integrate disturbance rejection capabilities with the fault-tolerant control framework and validate the enhanced system through experimental verification on physical robotic fish platforms in real-world environments.

## Figures and Tables

**Table 1:** Fuzzy control rules of CPG frequency.

*freq*		$f_{u}$			
		ZE	PS	PM	PB
$v_{factor}$	ZE	ZE	PS	PM	PB
	PS	ZE	PS	PM	PB
	PM	ZE	PM	PB	PB
	PB	ZE	PB	PB	PB

**Table 2:** Fuzzy control rules of CPG bias.

Bias		$e_{\psi ,f}$						
		NB	NM	NS	ZE	PS	PM	PB
$r_{factor}$	NB	PM	PS	ZE	NM	NB	NB	NB
	NM	PS	PS	ZE	NS	NB	NB	NB
	NS	PS	PS	PS	NS	NM	NB	NB
	ZE	PB	PM	PS	ZE	NS	NM	NB
	PS	PB	PM	PS	ZE	NS	NS	NS
	PM	PB	PM	PM	PS	ZE	NS	NS
	PB	PB	PB	PM	PS	ZE	NS	NM

**Table 3:** RMSE comparison for different methods toward four targets.

RMSE (rad)	Target 1	Target 2	Target 3	Target 4
PID	0.443	0.442	0.442	0.444
Basic Fuzzy	0.431	0.432	0.428	0.432
Our method	0.015	0.015	0.019	0.015

**Table 4:** Fault parameters of different fault types for pentagram waypoint following.

Fault Type	I	II	III	IV	V
Fault Parameters					
Joint	1	2	3	4	2
Locking angle	−20°	25°	−35°	40°	30°

## Data Availability

The data generated during the current study are available from the corresponding author on reasonable request.
